# Ceramic-Integrated Eddy Current Sensor for Blade Tip Clearance Measurement: Design and Performance Evaluation

**DOI:** 10.3390/s26103101

**Published:** 2026-05-14

**Authors:** Qiang Miao, Zhichun Liu, Qijian Liu

**Affiliations:** School of Aerospace Engineering, Xiamen University, Xiamen 361102, China; miaoqiang@stu.xmu.edu.cn (Q.M.); liuzhichun@xmu.edu.cn (Z.L.)

**Keywords:** blade tip clearance measurement, eddy current sensor, high temperature tolerance, ceramic integration

## Abstract

Blade tip clearance (BTC) is a critical parameter for the thrust, fuel consumption, and operational safety of aero-engines, and its accurate monitorinfg is of significant engineering importance. Traditional eddy current sensors (ECS) in BTC measurement often employ wound coil structures, which suffer from issues such as poor consistency and limited geometric shapes, restricting further optimization of electromagnetic performance. This paper proposes a novel ECS based on ceramic-integrated printed coils. The ECS uses screen printing technology to directly print metal coils onto ceramic substrates and integrate them into a single unit, allowing the coils to be designed with high precision into any topology structure, with high consistency, structural stability, and high temperature tolerance. Performance studies indicate that the sensor can be manufactured with an accuracy of 0.2 mm or better, and the sensor with a line width and spacing of 0.2 mm performed the best in the test. Not only does it exhibit the best electromagnetic performance at room temperature, but it also shows an electromagnetic performance variation of less than 1% after a 24 h aging test at 800 °C. Additionally, it provides stable peak-to-peak and periodic responses to changes in BTC within the range of 0 to 600 rpm for the fan motor. This study provides a promising method for accurate and stable BTC measurement at high temperatures.

## 1. Introduction

Aero-engines are widely regarded as the “jewels in the industrial crown” and serve as key indicators of national technological and industrial capability. Rotor blades, as the core power-generating components of engines, directly affect the operational status, efficiency, and safety performance of the entire engine system through their own operational parameters [1]. Notably, the tip clearance parameters between the rotor blade tips and the inner wall of the engine casing are directly related to engine efficiency, pressure ratio, fuel consumption rate, stability, and other factors, making them crucial for enhancing engine performance [2]. Studies have shown that in turbine stages, approximately one-third or more of the aerodynamic losses are caused by tip clearances, and for every 1% increase in the tip clearance relative to the length of the turbine blade, the aerodynamic efficiency of the turbine decreases by 0.8% to 1.2% [3]. Therefore, blade tip clearance (BTC) measurement and control is of great significance for preventing engine safety accidents, improving engine efficiency, and reducing engine maintenance costs.

The prevalent non-contact measurement methods for the tip clearance currently include the optical fiber method [4,5], capacitive method [6,7], laser method [8,9], microwave method [10,11], and eddy current method. Among them, the optical fiber method, despite its high measurement accuracy, is sensitive to contamination and is mostly suitable for clean and low-temperature environments. Although the capacitive method exhibits good high temperature tolerance, its measurement range is limited, and its output is prone to drift due to environmental interference. The laser and microwave methods, on one hand, perform excellently under ideal laboratory conditions; however, their system costs are extremely high, limiting their widespread application. The eddy current method, based on the principle of electromagnetic induction, operates as follows: when the blade tip passes by the sensor, the magnetic induction lines around the induction coil cut by the blade generate induced eddy currents. These induced currents modify the coil impedance through changes in the mutual inductance between the blade and the sensor. By detecting this impedance change, the tip clearance value can be calculated. Therefore, the eddy current sensor (ECS), with its core component being only an induction coil, possesses significant advantages such as simple structure, strong tolerance to contamination, and good environmental adaptability. It is particularly suitable for extreme operating conditions such as high temperature, high pollution, and strong vibration present at the turbine end of aero-engines. Therefore, it holds an irreplaceable position in the field of tip clearance monitoring.

Numerous studies have been conducted by scholars on the application of the ECS in BTC measurement. Chana et al. [12,13] developed a high-temperature ECS capable of withstanding temperatures ranging from 1500 to 1600 K, and has completed practical verification tests on engines. Jiang et al. [14] developed a high-performance ECS with a resolution of 10 μm, achieving tip clearance measurement of turbine rotors under conditions of 1300 K high temperature and 30,000 rpm. Wu et al. [15] proposed a speed adaptive adjustment model to address the issue of signal attenuation at high rotational speeds, and in 2021, further proposed a measurement method based on event capture technology, requiring only 0.1% of the hardware resources needed for continuous measurement. Liu et al. [16,17] achieved an ECS capable of withstanding 1100 °C, and has successfully measured the tip clearance of a test motor with a blade width of 1.8 mm at 1000 °C.

Existing research has primarily focused on measurement methods, high temperature tolerance characteristics, and engineering applications, whereas investigations into the optimization of sensor structural configurations remain comparatively limited. This is closely related to the current mainstream packaging structures. Currently, sensors mostly employ high-temperature-tolerant metals such as platinum wire to wind planar spiral coils, which are encapsulated in ceramic or high-temperature alloy housings [18]. Although these structures can meet basic needs, they still have the following limitations. Firstly, the process consistency is poor, and even with the same process, the consistency of sensor parameters is still difficult to guarantee. Secondly, the coil topology is limited to planar spiral shapes, making it difficult to achieve complex configurations such as multi-layer and overlapping structures, which restricts the further improvement of electromagnetic performance. For example, while a traditional ECS can hardly achieve a topology design with multiple line widths and spacings within a single sensor, this is readily achievable using the ceramic-integration method proposed in this paper. Lastly, thermal mismatch and fretting wear are prone to occur between the coil and packaging materials, affecting the stability of output signals and the long-term reliability of the sensor.

Addressing the aforementioned issues, this paper proposes a novel ECS based on ceramic-integrated printed coils. This approach involves directly printing the induction coil onto a ceramic substrate using screen printing technology and then co-firing it at high temperatures. This not only retains the advantages of high consistency and pattern complexity inherent to printing technology, but also significantly enhances the thermal stability and structural reliability of the sensor by leveraging the excellent high temperature tolerance, thermal shock tolerance, and insulating properties of ceramic materials. Based on this approach, the subsequent content of this paper will focus on the high-precision design and fabrication of sensors, optimization of electromagnetic performance, high temperature tolerance and BTC sensing performance, conducting experiments and discussions to evaluate the specific advantages of ceramic-integrated methods. The aim is to provide a feasible path for sensor standardization, high temperature tolerance and sensitivity to BTC measurement.

## 2. Materials and Methods

To prepare ceramic-integrated ECS suitable for high temperature, ensuring process consistency and reliability, the material selection for the sensors primarily considered ink, solder, and lead wire three types. Among them, the ink selected is 01H-6301 conductive silver paste (Shenzhen Saiya, Shenzhen, China), which is composed of high-purity silver particles, glass binder, and organic carrier. It has a temperature tolerance of up to 850 °C, possesses extremely low sheet tolerance in the milliohm range, and exhibits good tolerance to oil contamination, making it suitable for the preparation of ceramic-integrated sensors. The solder chosen is 01H-1803 conductive silver paste (Shenzhen Saiya, Shenzhen, China), which has a wide sintering temperature range and high bonding strength. Combined with the pressing disc process, it can achieve high-strength connection between the lead wire and the sensor, with almost no thermal loosening at high temperatures, effectively enhancing structural reliability. The lead wire is made of nickel wire, with a melting point of 1453 °C. Above 500 °C, it can form a dense oxide film, combining good oxidation tolerance and insulation properties, which helps to ensure the electromagnetic stability of the lead wire at high temperatures.

**Figure 1:**
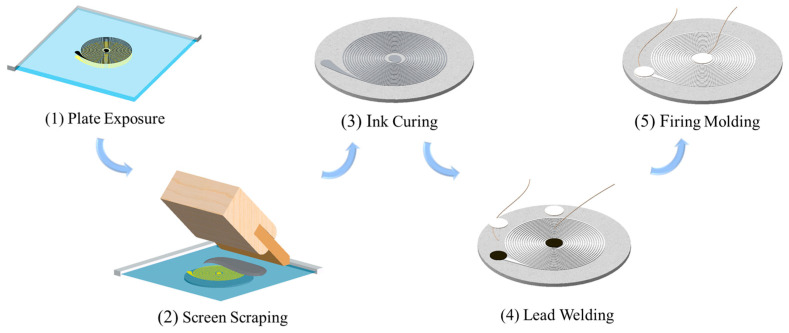
Flow chart of sensor fabrication.

This paper adopts screen printing as the printing process for ceramic-integrated printed coils. The overall printing process mainly includes five steps: exposure and plate making, screen printing, ink curing, lead wire soldering, and firing and molding. The specific manufacturing process is shown in Figure 1. The round ceramic substrate used for printing has a diameter of 40 mm and thickness of 1 mm. There are two solder joints with the diameter of 3 mm distributed at the center and edges of the substrate.

**Figure 2:**
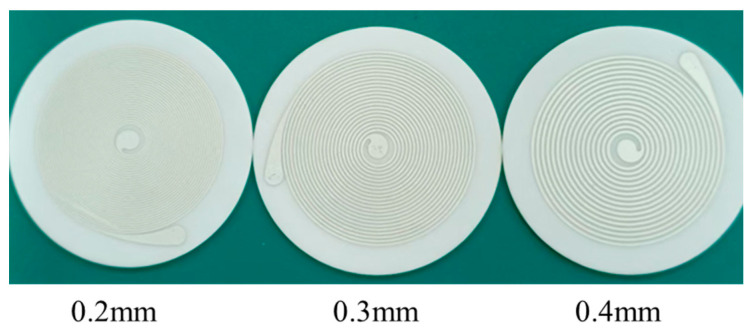
Comparison of sensor samples with different line widths.

The mesh size refers to the number of threads per inch of the screen used in screen printing, which has a crucial impact on the consistency and reliability of the printing process. A mesh size that is too low (100–200 mesh) can easily lead to rough lines and blurred edges, while a mesh size that is too high (above 400 mesh) can hinder the penetration of ink, making it difficult to control thickness and increasing tolerance. After experimental comparison, this paper selects a 300-mesh screen to print spiral patterns according to the process shown in Figure 1. After printing, the ceramic substrate coated with silver paste is placed in a heating furnace for curing and sintering, forming a dense silver-white coil structure. Three types of coils with diameters and spacings of 0.2 mm, 0.3 mm, and 0.4 mm are printed, as shown in Figure 2. The coil material and ceramic substrate have been fully sintered. All coils are inspected using a microscope after fabrication, and even the thinnest 0.2 mm printed coil exhibits high curing quality, indicating that the sensor process has good consistency and stability.

Although, in theory, a line width of 0.1 mm can be achieved through techniques such as increasing the mesh size of the screen, preliminary experiments indicate that this scale poses extremely high demands on printing positioning accuracy, paste uniformity, and process stability. When the line width is reduced to 0.1 mm, the probability of defects such as missed prints and misprints significantly increases, making it difficult to ensure the reliability and batch consistency of sensor fabrication. Related studies [19,20] also point out that the process yield of screen printing sharply decreases when the line width is below 0.15 mm. Therefore, this study concludes that a line width of 0.2 mm is close to the feasible limit under current process conditions.

## 3. Electromagnetic Performance Experiments

Unlike conventional wound coils, the ceramic-integrated ECS features a non-tightly wound structure, meaning that adjacent printed turns have no inter-turn insulation and cannot be tightly packed like enameled wires. Therefore, the inter-turn spacing plays a critical role in the electromagnetic performance. Research [21] shows that for non-tightly wound spiral induction coils, increasing the line width or number of turns can help improve inductance and electromagnetic performance. This chapter details the experimental verification conducted on ceramic-integrated coils based on this rule.

In order to explore the effect of geometric parameters of ceramic-integrated ECS and provide a basis for the structural optimization of new sensors, this study prepared five ceramic-integrated printed sensors with different combinations of line width and turns. The specific parameters are shown in Table 1. Due to the different wire diameters and distances used, the number of turns of different coils is constrained by the position of solder joints. The specific principle is to select the maximum number of turns without exceeding the position of solder joints. The distribution of solder joints is shown in Figure 1 and Figure 2 mentioned above. All coils have an outer diameter of approximately 35 mm and an inner diameter of 5 mm because of the fixed solder-joint positions.

**Figure 3:**
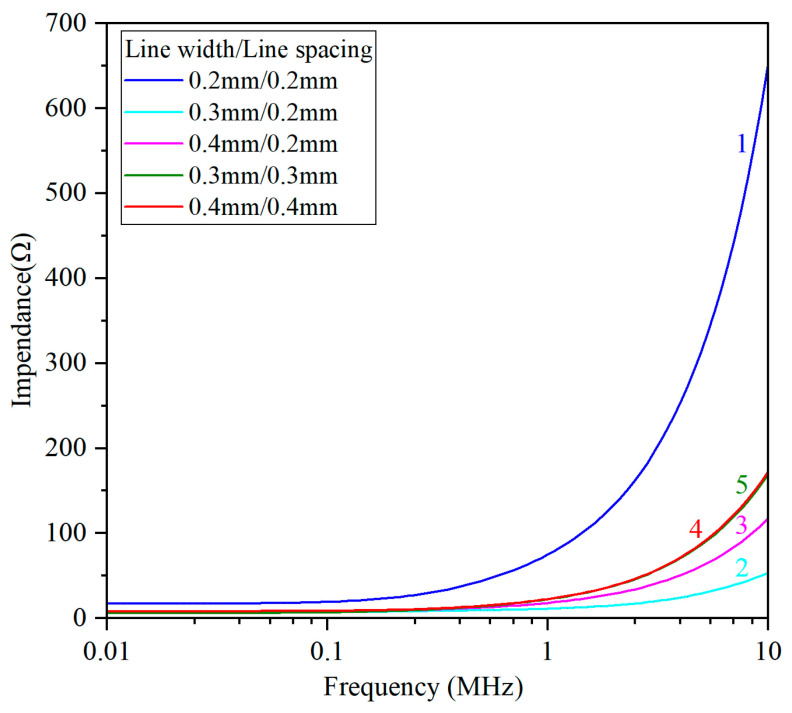
Comparison of response of five sensors with different parameters.

In order to evaluate the eddy current detection performance of the sensor, this paper uses impedance analyzer WK6500B (Wayne Kerr Electronics, London, UK) to compare the frequency-impedance response curves of the same sensor. The test results of the five sensors are shown in Figure 3.

The eddy current detection performance of sensors can be reflected by the steepness of the frequency-impedance curve. This is because when the blade passes through the sensor, the electromagnetic interaction occurring in the sensor will cause the coil to induce frequency components related to the blade motion. The steeper the frequency-impedance curve of the coil, the larger the range of impedance variation in the blade motion signal, and the more obviously the modulation signal can be detected. As shown in Figure 3, within the frequency range of 0.01~10 MHz, the impedance of each sensor shows an upward trend with increasing frequency, and the curve grows exponentially, which is consistent with the typical frequency characteristics of inductive components. By comparing sensors with different combinations of line width and number of turns, the following can be concluded:

(1) Under the same line width conditions, the frequency-impedance curve of sensor (Sensor 1) with a line spacing of 0.2 mm is the steepest, and its electromagnetic performance is significantly better than other sensors. However, the electromagnetic performance of sensors with a line spacing of 0.3 mm (Sensor 4) and 0.4 mm (Sensor 5) are relatively similar.
(2) When the line spacing is fixed at 0.2 mm, the effect of line width on sensor performance is not monotonic. The electromagnetic performance of the sensor with a line width of 0.3 mm (Sensor 2) is inferior to that of the sensor with a line width of 0.4 mm (Sensors 3). This indicates that there is a matching relationship between line width and number of turns, rather than thinner line width and more turns resulting in better electromagnetic performance.
(3) The impedance characteristics of sensors with equal line width and line spacing (Sensors 1, 4, 5) are generally better than those of sensors with unequal line width and line spacing (Sensors 2, 3), indicating that equal line width and line spacing have a positive impact on electromagnetic performance.

In fact, the space on the ceramic substrate is very limited, and an increase in the 0.1 mm line width will have a significant impact on the overall number of turns of the sensor. In order to achieve excellent electromagnetic performance while maintaining sensor miniaturization, the strategy of smaller line width and more turns is significantly better than the strategy of larger line width and fewer turns, under the premise of equal wire diameter and wire spacing. Meanwhile, the phenomenon of matching between wire diameters has been studied in the production of printed circuit boards with the similar structure [22,23], which suggests that changes in wire spacing can affect the coupling capacitance between coil turns.

In conclusion, not only should the coil shape be refined, but also the coupling caused by non-dense windings should be considered to achieve optimal coil performance.

## 4. Temperature Degradation Experiments

**Figure 4:**
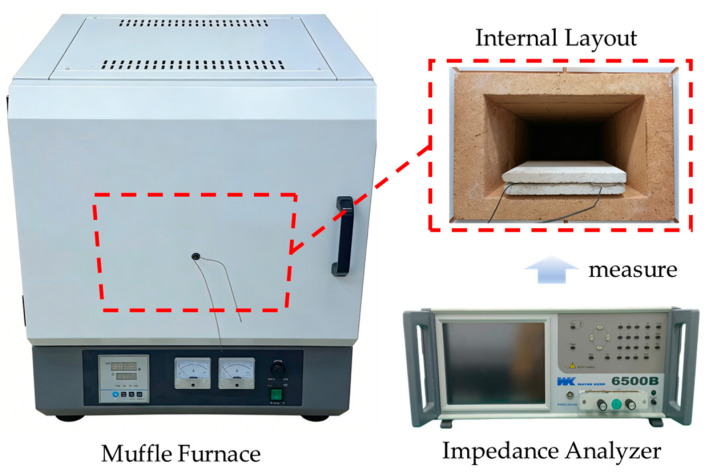
Experimental setup for temperature loading.

To test the high temperature tolerance performance of the sensor, a sensor sample with a line width of 0.2 mm was selected for temperature loading and degradation experiments. Before the experiment, nickel wires with a diameter of 1 mm were welded at both ends of the sensor as leads, and high-temperature insulation paint was applied to ensure insulation performance at high temperatures. The sensor was clamped between two mullite bricks and placed in a muffle furnace for temperature loading. The experimental setup is shown in Figure 4.

**Figure 5:**
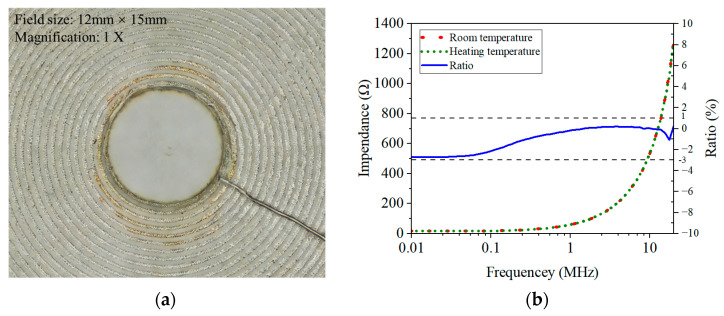
Temperature degradation experiment. (**a**) Microscopic magnification of sensor solder joints after experiment (**b**) Frequencyimpedance response curves before and after experiment.

The temperature degradation experiment was conducted as follows: Raise the muffle furnace from room temperature to 800 °C within 1 h, and then maintain the heating furnace at 800 °C for 24 h to conduct a thermal aging heating test on the sensor. During the thermal aging heating process, the impedance analyzer WK6500B was used to apply AC excitation of 1 MHz and 1 Vpp to the sensor throughout the entire process. The microscopic magnification of sensor solder joints after experiment is shown in Figure 5a. After 24 h of aging testing, the sensor turned fully yellow due to the heating effect of the silicon carbide rods rods in the muffle furnace, but there were no signs of high-temperature damage overall. As shown in Figure 5a, there is no disconnection or blurring of the printed wire, and the joint between the lead and the sensor is still very firm. To investigate the performance degradation of the sensor after long-term high-temperature use, its frequency impedance response curve was subsequently remeasured and compared with the curve before heating.

The aforementioned experiments fully demonstrate that the material system and microstructure design employed in the ceramic-integrated sensor are capable of enduring long-term high-temperature service and possess excellent potential for engineering applications. Moreover, it was observed that the electrical parameters of the sensor exhibited significant temperature sensitivity and a certain degree of time delay during the temperature load process. The specific temperature drift is complex, and it is difficult to compensate directly using the current sensor structure alone.

## 5. Dynamic BTC Monitoring Experiment

**Figure 6:**
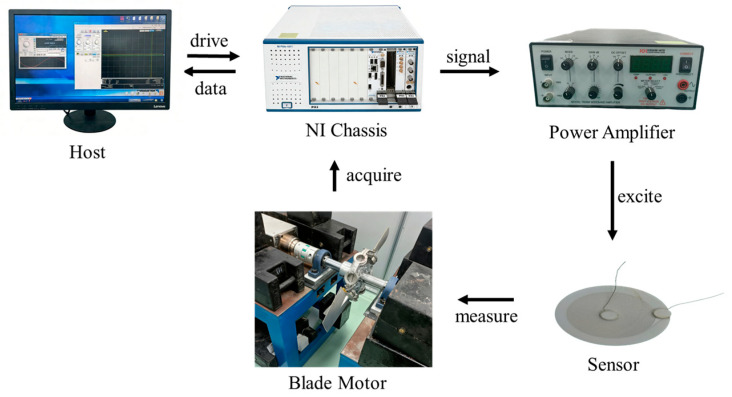
Experimental system for blade tip clearance measurement.

To evaluate the BTC measurement performance of the sensor, this paper establishes a dynamic monitoring test platform for fan blades, with the system shown in Figure 6. The platform mainly includes a chassis NI-PXle-1071 (National Instruments, Austin, TX, USA), a power amplifier Krohn-Hite® KH-7600M (Krohn-Hite Corporation, Brockton, MA, USA), a sensor sample and a fan motor. The NI-PXle-1071 integrates an NI-5442 (National Instruments, Austin, TX, USA) signal generator and an NI-5122 (National Instruments, Austin, TX, USA) acquisition card, with a resolution of 13 bits and a maximum sampling rate of 100 MS/s. The power amplifier increases the power of the sensor excitation signal and stabilizes the output voltage. The fan motor used in the experiment has a diameter of 60 cm and is equipped with two wing-shaped aluminum alloy blades; the maximum stable rotation speed for the fan motor is 600 rpm. The sensor is placed directly below the shaft, 3 mm away from the blade tip.

**Figure 7:**
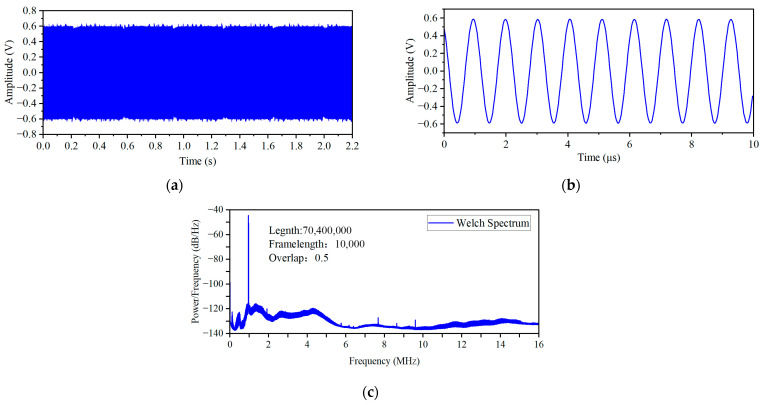
Time domain and frequency domain information of the original signal. (**a**) Total 2.2 s waveform (**b**) First 10μs waveform (**c**) Welch spectrum of signal.

To achieve a balance between acquisition quality and duration, the excitation signal was determined to be a 1 MHz, 1.2 Vpp sine wave after debugging, and the sampling frequency was set to 32 MHz. With this configuration, 32 points can be sampled per excitation cycle, far exceeding the minimum requirement of the Nyquist sampling theorem. The original signals obtained by acquiring a 2.2 s waveform at a fan speed of 100 rpm are shown in Figure 7a,b. The local waveform of the acquired signal is complete with minimal distortion, indicating high quality in the time domain acquisition. However, overall, although the original signal exhibits certain gap characteristics, the background noise is still evident. Directly calculating the frequency spectrum of the entire data segment would involve a large amount of computation, so the Welch power spectrum estimation method was adopted to perform spectral analysis after applying a Hanning window to the signal. The frame length was set to 10,000 points, with an overlap rate of 50% between frames. The analysis result is shown in Figure 7c. From Figure 7c, it can be seen that the amplitude of the 1 MHz excitation frequency component is much higher than that of other frequency components, indicating that the acquired signal has an extremely high signal-to-noise ratio.

**Figure 8:**
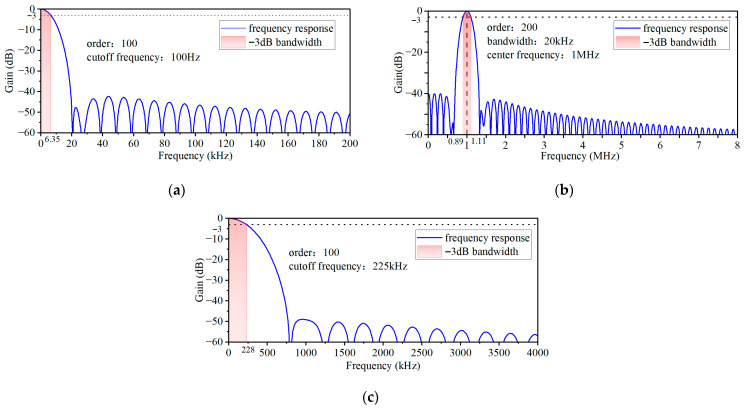
Parameters and frequency responses of filters: (**a**) band-pass filter; (**b**) anti-aliasing filter; (**c**) low-pass filter.

To achieve gap signal extraction, this paper adopts a signal processing strategy combining multiple filters. Firstly, the 1 MHz fundamental frequency component is extracted through a bandpass filter. Subsequently, the filtered signal undergoes a 640-fold downsampling and is paired with anti-aliasing filtering to reduce the data volume and suppress clutter. Then, the Hilbert transform is employed to extract the signal envelope. Finally, the envelope undergoes smoothing through low-pass filtering. To enhance the processing effect, the parameters of each filter stage have been optimized. The final parameters and frequency response are shown in Figure 8.

**Figure 9:**
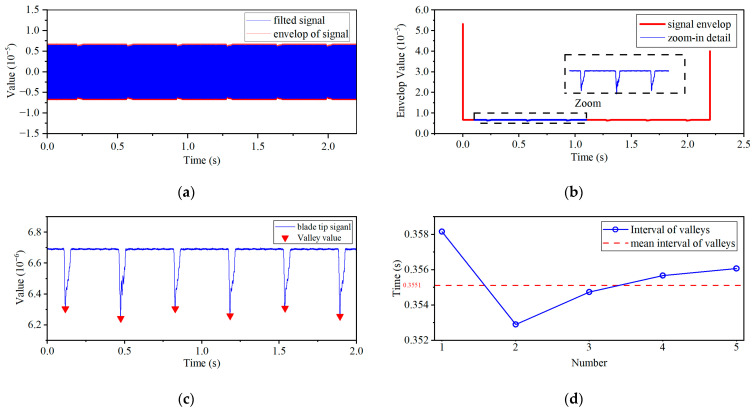
Envelope extraction and truncation and valley detection of tip clearance signal. (**a**) Hilbert transform. (**b**) Signal after low-pass filtering. (**c**) Result after truncation and valley extraction. (**d**) Valley interval statistics.

After anti-aliasing filtering, the noise unrelated to the gap is basically eliminated, and the signal envelope begins to emerge, as shown in Figure 9a. After further smoothing through low-pass filtering, although there are abnormal values introduced by the calculation at the beginning and end of the signal envelope, the gap signal is overall clear and distinguishable, as shown in Figure 9b. After truncating the beginning and end of the envelope signal by 0.1 s each, the final gap waveform is obtained, as shown in Figure 9c. The valley values of the gap waveform are extracted using peak extraction algorithm based on local extremum to obtain the tip clearance detection results of the ECS at 100 rpm, as shown in Figure 9d.

**Figure 10:**
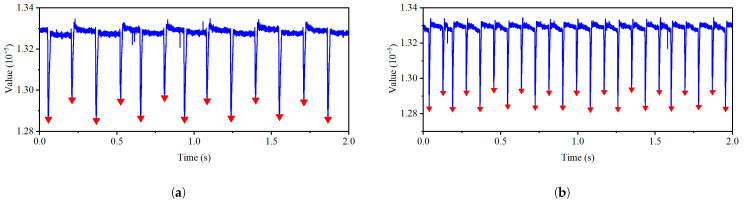

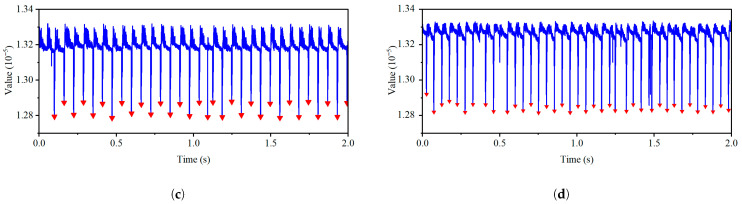
Extracted tip clearance signals at different rotational speeds. (**a**–**d**) Results at speeds of 210, 350, 500, and 600 rpm.

As can be seen from Figure 9, when the motor speed is 100 rpm, the blades passing by the sensor can be detected clearly and stably, forming two sets of periodic spikes with slightly different heights. Based on the same data processing method, experiments were further conducted on the BTC signals at speeds of 210 rpm, 350 rpm, 500 rpm, and 600 rpm. The clearance waveforms at each speed are shown in Figure 10. From Figure 10, it can be seen that as the speed increases, the monitoring signal periods corresponding to the two blades shorten accordingly, and the peak shapes gradually become compact. At 600 rpm, the signal quality of the BTC decreases, but the monitoring waveform still exhibits stable periodic characteristics. The trough information at each speed is summarized in Table 2.

Based on the valley detection results shown in Figure 10 and Table 2, the sensor exhibits stable response characteristics to the BTC waveform within the speed range of 100 to 600 rpm. As the speed increases from 100 rpm to 600 rpm, the number of valleys increases from 6 to 38, which is consistent with the theoretical expectation that the blade passing frequency (blade number × speed/60) increases linearly with speed, thus verifying the dynamic response capability of the sensor. The average interval shortens from 0.3555 s to 0.0528 s, showing a linear relationship with the inverse of speed (1/rpm), further confirming the reliability of the measurement results. The above results indicate that the sensor has good sensing capability for BTC within the measured speed range, demonstrating its potential for engineering applications under dynamic conditions.

## 6. Conclusions

This article proposes a novel ECS based on ceramic-integrated printed coils, which meets the requirements of high temperature tolerance, high-precision manufacturing, and BTC monitoring capability. This article presents a systematic full-chain study demonstrating the potential application of BTC measurement in aero-engines. The main conclusions are as follows:

(1) A new method for preparing ceramic-integrated sensors with high consistency and design freedom has been proposed, achieving high-precision integration of coils and substrates. This method can achieve high-precision coil production with a resolution of not less than 0.2 mm. It not only solves the problem of poor consistency in the winding of ECS used for BTC measurement, but also breaks through the local limitation of the original winding sensors that can only adopt limited topology dense winding, freeing up the design space of coil topology structure.
(2) This study revealed the influence of two geometric parameters, line width and line spacing, on the electromagnetic performance of non-tightly wound structures of printed coils. The results show that there is a coupling relationship between the line width and line spacing of the coil, and using a design with equal line width and line spacing is beneficial for improving the electromagnetic performance of the sensor. Meanwhile, while ensuring the miniaturization of sensors, adopting a refined strategy is beneficial for improving the electromagnetic performance of sensors. This discovery provides a basis for the subsequent basic topology design and optimization of sensors.
(3) The study verified the excellent stability and engineering application potential of ceramic-integrated sensors. After conducting a 24 h aging experiment at 800 °C, the frequency-impedance curve of the sensor changed by less than 1% within the core operating frequency range (1–10 MHz), indicating excellent long-term thermal stability of its material system and microstructure. In the dynamic monitoring test of fan blades, the sensor effectively responded to BTC waveforms within the speed range of 100 to 600 rpm, fully demonstrating the solid engineering application potential of the sensor.

In summary, this paper systematically demonstrates the technical feasibility of using ceramic-integrated ECS for BTC measurement from four aspects: manufacturing process, electromagnetic response characteristics, high temperature tolerance, and dynamic performance evaluation.This study provides a new technological path for improving the high temperature tolerance and vibration resistance of sensors, thereby achieving accurate and stable measurement of BTC.

This paper has fully confirmed the feasibility and application potential of a ceramic-integrated printing scheme in BTC measurement. Subsequent research will be focus on three directions: material and process optimization, dynamic temperature drift compensation, and high-speed fan BTC measurement. The topology advantages of ceramic-integrated design will be fully utilized to develop sensors with higher sensitivity and more in line with engineering reality.

## Figures and Tables

**Table 1:** Sensor parameters with different line width-turn combinations.

Number	Line Spacing/mm	Line Width/mm	Number of Turns
1	0.2	0.2	32
2	0.2	0.3	26
3	0.2	0.4	22
4	0.3	0.3	22
5	0.4	0.4	16

**Table 2:** Statistics of valley detection.

Number	Rotation Speed/rpm	Number of Valleys	Average Interval/s
1	100	6	0.3555
2	210	13	0.1508
3	350	23	0.0871
4	500	32	0.0611
5	600	38	0.0520

## Data Availability

The original contributions presented in this study are included in the article.

## Abbreviations

The following abbreviations are used in this manuscript:

BTC	blade tip clearance
ECS	eddy current sensor
